# Rarity, Species Richness, and the Threat of Extinction—Are Plants the Same as Animals?

**DOI:** 10.1371/journal.pbio.1001067

**Published:** 2011-05-24

**Authors:** Sandra Knapp

**Affiliations:** Department of Botany, The Natural History Museum, London, United Kingdom

## Abstract

Assessment of conservation status is done both for areas or habitats and for species (or taxa). IUCN Red List categories have been the principal method of categorising species in terms of extinction risk, and have been shown to be robust and helpful in the groups for which they have been developed. A recent study highlights properties associated with extinction risk in flowering plants, focusing on the species-rich hot spot of the Cape region of South Africa, and concludes that merely following methods derived from studies of vertebrates may not provide the best estimates of extinction risk for plants. Biology, geography, and history all are important factors in risk, and the study poses many questions about how we categorise and assess species for conservation priorities.

Conservation of life on earth has become much more than a side interest of a few scientists and is now part of mainstream international activity, largely through the Convention on Biological Diversity (CBD), first ratified in 1992. The CBD has three aims: 1) conservation of biological diversity, 2) sustainable use of its components, and 3) fair and equitable sharing of the benefits arising out of the utilisation of genetic resources [1]. The primary focus of the CBD, therefore, is the fair and sustainable use of biodiversity, but its conservation comes first, and with the ecosystem services framework developed as part of the Millennium Ecosystem Assessment [2], it is clear that biodiversity is essential for the delivery of these services, but just how this works is not completely clear. The CBD 2010 targets for reversing the rate of biodiversity loss were famously not perfectly met [3], but good progress was made towards developing new indices for monitoring the degree to which our species, *Homo sapiens*, manages to preserve the diversity of life on earth that underpins our own well-being [4],[5]. Biodiversity itself is a relatively new concept—it can be thought of as the diversity of life on earth at all levels, from genes to ecosystems. This includes species threatened with extinction that have been the targets of conservation action for decades.

Since the recognition of the effect human alteration of habitat has been having on the rest of the species with which we share the planet, many different methods of assessing both diversity and threat have been established and supported. Hot Spots [6], Important Bird (and Plant) Areas (http://www.birdlife.org/action/science/sites/index.html, http://www.plantlife.org.uk/international/campaigns/IPA/ipa_online_database/), and the Global 200 [7] are among the many categorisation methods or systems that have been proposed as good relative importance measures for global area conservation. All of these have species diversity (usually as numbers of species) as one important component of their definition, thus species themselves are important. In recent years, the use of only species numbers (richness) as a measure of biodiversity has been augmented with the use of phylogenetic diversity; these measures take into account the evolutionary relatedness of the species in an assemblage (reviewed in [8]). These measures basically contend that an assemblage containing more phylogenetically divergent species is more important than one in which all the species are closely related. So, to use an extreme example, a piece of woodland with a bluebell, a robin, and a bear would be more “valuable” than one with a bluebell, a daffodil, and a daisy; but as you might have noticed by now, missing species certainly might matter in how measures of phylogenetic diversity are estimated. If these hypothetical lists do not represent complete inventories or complete phylogenies, then any measure of value using them is nonsense. Measures combining phylogeny and abundance and/or range size can be used to better quantify and predict diversity and relative conservation importance of sites (e.g., [9],[10]).

At the species level, assessments of conservation status are generally performed using the International Union for Conservation of Nature (IUCN) Red List criteria, first published in 1994 [11], and both the categories and criteria have been reviewed and improved several times since [12]. The categories used in the Red List range from Least Concern (LC) to Extinct in the Wild (EW) to Extinct (EX), and include a category of Data Deficient (DD) in order to highlight taxa for which information is not sufficient to make a sound assessment of status. The five criteria for evaluation are 1) declining population (past, present, or future), 2) a measure of geographic range (including fragmentation, decline, or fluctuation), 3) small population size and fragmentation, decline, or fluctuation, 4) very small population size or very restricted distribution, and 5) a quantitative analysis of extinction risk; in order to list a species, only one of these five need be met [13], but all should be considered.

The overwhelming majority of listed species are vertebrates from terrestrial ecosystems (see http://www.iucnredlist.org/); the Red List, however, has become a powerful and widely used tool in conservation [14]. The vertebrate bias is recognised by those managing the Red List, and great efforts are being made to expand the scope of the List taxonomically (see [15]). Evolutionary relatedness (or phylogeny) has also been suggested as an important factor for consideration in setting priorities for species-level conservation [16]. An index (evolutionary distinctiveness, or ED) combining phylogenetic diversity and IUCN Red List categories has been developed that sets species-level priorities for conservation [17]; this has shown that species with low ED scores are also those at less risk of extinction (as measured by their IUCN category). EDGE (evolutionarily distinct globally endangered) scores have been calculated for a variety of vertebrates and for corals (see http://www.edgeofexistence.org/), and in general, species with few living relatives also tend to be those most at risk of extinction.

Most (but not all) of the indicators of biodiversity status are based on vertebrates, but most of the life on earth is invertebrate or micro-organismal by orders of magnitude. Do the criteria for setting conservation priorities or assessing risk of extinction at the species level that have been developed for vertebrates really work for other taxonomic groups? Can we predict extinction risk from biological traits? Are the species that have been listed really those most at risk? In Europe at least, it appears that this last scenario is not the case [18]. The paper by Davies et al. [19] in this issue of *PLoS Biology* suggests that in fact, for plants, the measures developed for vertebrates may provide misleading indications of extinction risk. To do this, the authors used an amazing dataset for flowering plants that comprised a complete Red List assessment for all taxa from two regions, the Cape floristic region and the United Kingdom, and a complete phylogeny at the generic level of the Cape flora. The Cape is one the most species-rich areas on the globe for flowering plants, and the flora has extraordinarily high endemism, suggesting *in situ* diversification, while the flora of the United Kingdom has been assembled by post-glacial recolonisation and range expansion. These two very different assemblages are not only excellent for comparison but in fact are the only such datasets for plants anywhere. If key traits are linked to extinction risk, then the signal should be detectable in both of the regions, and the idea that particular traits (life history, pollination syndrome, etc.) predispose plant species to extinction would be supported. An observed trend for rare plant species to be in species-rich lineages [20] suggests that speciation and extinction may be linked. If this is indeed true, there is no better place to detect this than in the Cape flora, which is full of rare species and where a number of complete species-level phylogenies for clades that have diversified within the region have been constructed.

In fact, the taxa at risk (analysing at the level of family) are different in the two regions, which is hardly surprising, as geography is probably as important as biology in making plant species (or any species, for that matter) vulnerable. The finer level of detail in the Cape flora dataset allowed more fine-grained analysis of this general pattern, and found only weak evidence for closely related lineages to contain similar proportions of species at risk; not at all what might be expected if particular traits predicted threat. What did matter in genera endemic to the Cape was the species richness of the lineage to which they belonged, and the age of that lineage. This analysis shows that the observed link between lineage richness and risk of extinction is the result of both richness and risk co-varying with lineage age; younger lineages have diversified faster so are species-rich, but a high proportion of these species are threatened with extinction. In short, threatened species are more common in lineages that are young and diversifying quickly. Quite a different result from that obtained for mammals [17].

Taking the analysis to a finer level in order to explore why this might be, disparity through time (DTT) analyses were done using 11 endemic Cape clades for which near-complete species-level phylogenies are available. These clades ranged from genera of orchids to tribes of sedges, but all are monophyletic and had very high species representation. DTT analyses are often used to track the tempo and mode of evolutionary radiation, the combination of diversification, and morphological and/or ecological change. The tempo and timing of change can be traced through time in both extinct [21] and extant lineages [22]. Modelling rates of change can be tricky, as the null model used can be unrealistic and extinctions can cause problems [23]; however, using these analyses in a phylogenetic context can mitigate these problems [24]. The DTT analyses done on these 11 clades from the Cape flora are a bit different—rather than using morphology as do most, a continuous linear scale was developed for risk using the IUCN categories from LC to EW. The variance in risk was partitioned between and among clades using two models, one Brownian (a random walk) and another punctuated (where risk was assigned asymmetrically to sister taxa). Two common trends emerged. Variation in risk was highest between species at the tips of the clades, and towards the root of the tree risk was conserved within clades. How peculiar. If risk is conserved in lineages, but differs wildly at the tips of those lineages, what is going on?

Davies et al. [19] suggest that mode of speciation lies at the root of this result; if plants speciate via small isolated populations at the edges of larger species ranges, then lineages that are diversifying rapidly will have larger numbers of threatened species, since range size is important for the assignment of IUCN status. In addition, if plants diversify predominantly through peripatric means—edge isolates—then the high variation in threat between the tips can also be explained. This pattern of widespread species with peripheral sister species of restricted distribution was remarked upon by Darwin [25] as common in plants; he predicted that widespread species would be more variable and in effect act as species pumps.

So maybe plants are different than animals, at least vertebrates, in terms of the predictability of extinction risk. But perhaps, too, the risk of extinction is really the result of human intervention in natural habitats and we don't need to worry about differing biology. Surprisingly, in the Cape flora dataset no correlation between anthropogenic transformation and threat was found. So, these threatened species in rapidly diversifying lineages are just intrinsically threatened; extinction and speciation both seem to be rapid. Small range size can mean that these young threatened species are going to expand and become less at risk, but the opposite seems to be true; species that are at risk are becoming more at risk through time (comparing Red Lists from different years). The authors suggest that hot spots are thus both cradles and graves of diversity—a disturbing metaphor.

The Cape flora, with its high levels of endemism and restricted habitat, may not be typical of plant assemblages, but the patterns explored in Davies et al. [19] cannot be ignored. Missing species, including species that are already extinct, in a phylogeny may make a lot of difference; datasets with which to compare these results will be a long time in coming. Few places on earth can boast such generic level endemism. Different regions may also exhibit distinct geography that patterns risk differently than the Cape; the Andean region, for example, has many isolated valleys with distinct biomes that harbour ancient endemics that can go nowhere, in addition to areas where rapidly diversifying lineages occur [26]. Risk signature through time would be interesting to explore in these floras, but lower generic level endemism could make this more difficult. It may be that restricted range, peripheral species are always doomed because they cannot expand their ranges easily; they may be on the edge of suitable habitat.

It is clear that restricted range species are disproportionately at risk of extinction, in both plants and animals. Rarity, however, can be thought of in a variety of different ways, not just in terms of range size. Rabinowitz [27] suggested that species become rare (and by extension subject to extinction risk) by a variety of pathways, and if this were so, the ecological and evolutionary consequences of rarity would be diverse. Her scheme took into account range size, habitat specificity, and local abundance (population size), and she used this to discuss the ecological consequences of rarity in terms of competition and co-existence, and the selective pressures likely to face rare taxa of the different sorts. Range size is important, but biology also matters.

Davies et al. [19] have shown that if we want to maximise the conservation of the tree of life the automatic application of criteria developed using one taxonomic group may not be the best idea for another. I suspect insects will be more like plants than like vertebrates (see [28]), and there are a lot of insects! Regions of rapid, explosive, recent diversification like the Cape are among those selected by most as priorities for conservation; here, perhaps less threatened species may be as worthy of effort as those currently doomed to extinction anyway. It is also apparent that we may need to rethink our ideas about species with wide distributions. If we fragment the ranges of these species of “least concern”, then will they lose their ability to generate peripheral isolates and operate as species pumps? Widespread species could also be at considerable risk, and not protecting them in favour of restricted taxa may in fact cause more loss of evolutionary potential. Strategies for implementation of the Global Strategy for Plant Conservation [29] may well need to take the results of Davies et al. [19] into account; if we intend to manage for future diversification as well as for current status, evolutionary, rather than only ecological, timescales become important. The bottom line is that many factors matter – geography, history, and biology. There is clearly no silver bullet for setting priorities; a solid, well-researched, and documented science base for conservation is critical for its practical and successful implementation across all taxonomic groups.

## Abbreviations

CBD: Convention on Biological Diversity
IUCN: International Union for Conservation of Nature
